# Supplementary material for: PInteract: Detecting Aromatic-Involving Motifs in Proteins and Protein-Nucleic Acid Complexes
Source: Biomolecules. 2025 Aug 21;15(8):1204. doi: 10.3390/biom15081204 (PMC12384504; doi:10.3390/biom15081204)
Supplement: Supplementary file 1 [file biomolecules-15-01204-s001.zip › biomolecules-3804195-Supplementary Materials.pdf]

Supplementary Material

PInteract: detecting aromatic-involving motifs in proteins and protein-nucleic acid complexes

Dong Li, Fabrizio Pucci, Marianne Rooman

Computational Biology and Bioinformatics, Université Libre de Bruxelles, Belgium
and
Interuniversity Institute of Bioinformatics in Brussels, Belgium

Table of content

- Section S1. Relative frequencies of π-interactions in different datasets.
- Section S2. Distributions of α and β angles characterizing π interaction geometry in experimental structures.
- Section S3. Distributions of α and β angles characterizing π interaction geometry in AlphaFold-predicted structures.
- Section S4. Example of π-π chains in amyloid fibrils.
- Section S5. Running efficiency of PInteract.

Section S1. Relative frequencies of π-interactions in different datasets

Table S1: P-values associated to the t-tests of the relative frequencies of π interactions in the six datasets $\mathcal{D}_{\text{monomer}}$, $\mathcal{D}_{\text{homodimer}}$, $\mathcal{D}_{\text{heterodimer}}$, $\mathcal{D}_{\text{TCRpMHC}}$, $\mathcal{D}_{\text{AbAg}}$, and $\mathcal{D}_{\text{protNA}}$. These frequencies were computed as the number of interactions identified by PInteract divided by the total number of interactions in the protein or at the protein-protein, protein-DNA, or protein-RNA interface. For details on the frequency computations, see Table 1 and Figure 3 of the main manuscript.

	Cation-π	Amino-π	His-π	Sulfur-π	π-π
$\mathcal{D}_{\text{monomer}}$-$\mathcal{D}_{\text{heterodimer}}$	3.63×10^{-7}	0.12	1.33×10^{-7}	0.91	0.62
$\mathcal{D}_{\text{monomer}}$-$\mathcal{D}_{\text{TCRpMHC}}$	0.26	0.027	0.40	0.081	2.96×10^{-3}
$\mathcal{D}_{\text{monomer}}$-$\mathcal{D}_{\text{AbAg}}$	3.78×10^{-16}	1.51×10^{-12}	3.33×10^{-6}	9.33×10^{-5}	1.94×10^{-2}
$\mathcal{D}_{\text{heterodimer}}$-$\mathcal{D}_{\text{TCRpMHC}}$	0.619	0.0535	0.0812	0.0819	3.06×10^{-3}
$\mathcal{D}_{\text{heterodimer}}$-$\mathcal{D}_{\text{AbAg}}$	3.20×10^{-9}	7.57×10^{-9}	2.06×10^{-10}	2.72×10^{-4}	3.01×10^{-2}
$\mathcal{D}_{\text{TCRpMHC}}$-$\mathcal{D}_{\text{AbAg}}$	1.21×10^{-5}	0.773	0.308	0.948	0.125
$\mathcal{D}_{\text{monomer}}$-$\mathcal{D}_{\text{homodimer}}$	2.05×10^{-17}	4.01×10^{-22}	2.34×10^{-67}	3.31×10^{-10}	3.60×10^{-68}
$\mathcal{D}_{\text{homodimer}}$-$\mathcal{D}_{\text{heterodimer}}$	2.39×10^{-14}	5.43×10^{-6}	4.29×10^{-3}	2.94×10^{-2}	1.99×10^{-11}
$\mathcal{D}_{\text{homodimer}}$-$\mathcal{D}_{\text{TCRpMHC}}$	0.0402	6.72×10^{-3}	2.29×10^{-2}	2.94×10^{-2}	0.869
$\mathcal{D}_{\text{homodimer}}$-$\mathcal{D}_{\text{AbAg}}$	7.15×10^{-20}	3.59×10^{-17}	2.04×10^{-15}	9.70×10^{-7}	6.23×10^{-3}
$\mathcal{D}_{\text{homodimer}}$-$\mathcal{D}_{\text{protNA}}$	1.60×10^{-3}	4.12×10^{-7}	4.46×10^{-9}	1.08×10^{-4}	4.07×10^{-2}
$\mathcal{D}_{\text{monomer}}$-$\mathcal{D}_{\text{protNA}}$	4.41×10^{-3}	2.13×10^{-2}	7.48×10^{-6}	1.27×10^{-2}	0.303
$\mathcal{D}_{\text{heterodimer}}$-$\mathcal{D}_{\text{protNA}}$	2.17×10^{-2}	0.542	1.14×10^{-7}	2.77×10^{-2}	0.346
$\mathcal{D}_{\text{TCRpMHC}}$-$\mathcal{D}_{\text{protNA}}$	1.84×10^{-2}	7.45×10^{-2}	8.39×10^{-3}	0.346	4.63×10^{-2}
$\mathcal{D}_{\text{AbAg}}$-$\mathcal{D}_{\text{protNA}}$	0.588	9.56×10^{-8}	1.51×10^{-2}	8.50×10^{-2}	0.142

Section S2. Distributions of α and β angles characterizing π interaction geometry in experimental structures

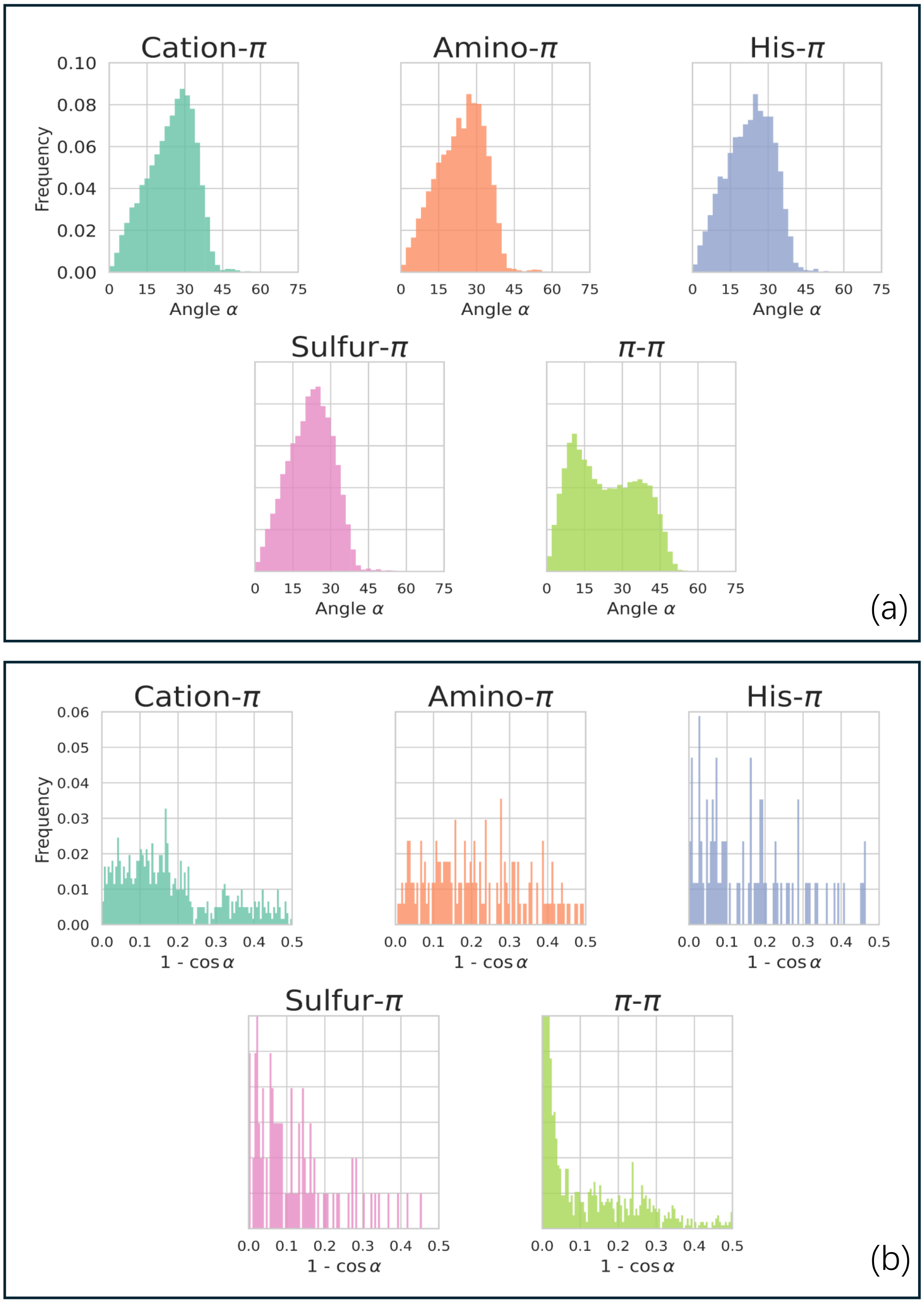

Figure S1: Distributions of α-angle values and of the lateral displacement $(1 - \cos\alpha)$, in which the α angle measures the location of the functional group of interacting partner 2 above (or below) the plane of the aromatic ring of partner 1, for different types of π interactions. (a) α-Angle distributions in $\mathcal{D}_{\text{monomer}}$; (b) $(1 - \cos\alpha)$ distributions in $\mathcal{D}_{\text{protNA}}$. The smaller the value of α, the more directly the functional group of partner 2 is positioned above (or below) the center of the aromatic ring. See Figure 5 of the main manuscript for details and the distributions of $(1 - \cos\alpha)$ in the $\mathcal{D}_{\text{monomer}}$ set.

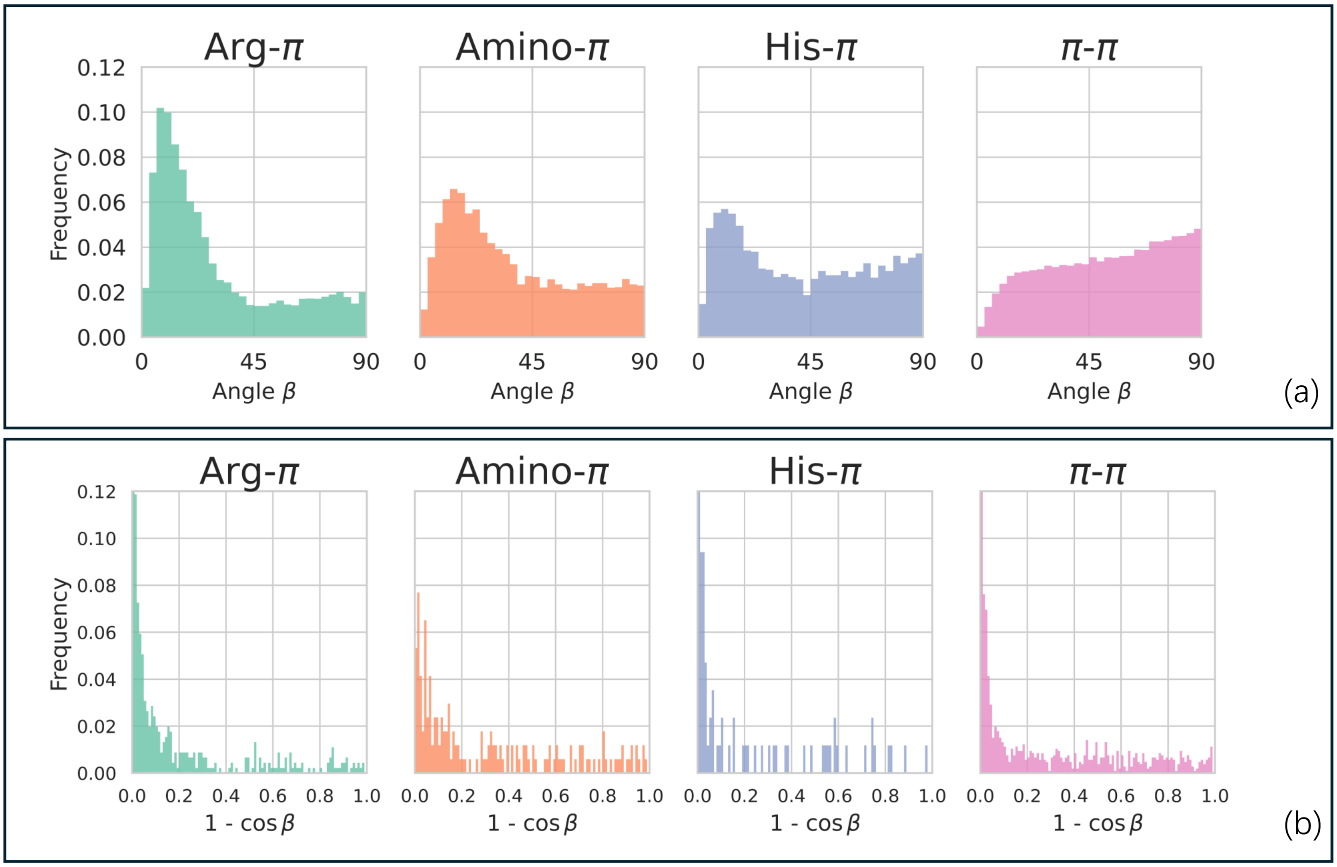

Figure S2: Distribution of β-angle and $(1-\cos\beta)$ values, in which the β angle measures the degree of parallelism between the interacting planar functional groups, for different types of π interactions. (a) β-Angle distributions in $\mathcal{D}_{\text{monomer}}$; (b) $(1-\cos\beta)$ distributions in $\mathcal{D}_{\text{protNA}}$. Parallel or stacked conformations correspond to $\beta = 0°$; perpendicular or T-shaped conformations, to $\beta = 90°$. See Figure 6 of the main manuscript for details and the distribution of the $(1-\cos\beta)$ values in $\mathcal{D}_{\text{monomer}}$.

Section S3. Distributions of α and β angles characterizing π interaction geometry in AlphaFold-predicted structures

To investigate whether the angular preferences of π interactions are also retained in computationally predicted structures, we applied PInteract to representative proteins from the AlphaFold Protein Structure Database (AlphaFoldDB) [47]. We assembled a dataset, denoted $\mathcal{D}_{\mathrm{AF}}$, comprising representative subsets of proteomes from four model organisms in AlphaFoldDB: *Arabidopsis thaliana*, *Escherichia coli*, *Homo sapiens*, and *Saccharomyces cerevisiae*. For each protein in $\mathcal{D}_{\mathrm{AF}}$, we run PInteract and extracted all π-involving interactions and the corresponding α and β angles as described in the main manuscript. The results are shown in Figure S3 and S4.

The distributions of α angles shown in Figure S3 are highly similar to those observed in experimentally determined monomer structures (see Figure 5 of the main manuscript), indicating that AlphaFold [48] tends to correctly predict π interaction geometries in which the interaction partner is located above (or below) the aromatic ring.

The β angle distributions depicted in Figure S4 also largely resemble those of experimental protein structures (see Figure 6 of the main manuscript), except for a subtle but systematic reduction of the frequency of stacked conformations for Arg-π, amino-π, and His-π interactions. In $\mathcal{D}_{\mathrm{AF}}$, stacked conformations are less representated than in experimental protein striuctures, whereas T-shaped confirmations are slightly overrepresented (Figure S4). This suggests that AlphaFold underestimates the energetic stabilization associated with stacked conformations in certain π-interactions.

Despite this minor difference, the general similarity between the angular distributions of π interactions in AlphaFoldDB and those observed in experimental protein structures supports the geometric realism of AlphaFold-predicted π interactions and further demonstrates the applicability of PInteract to computationally predicted structures.

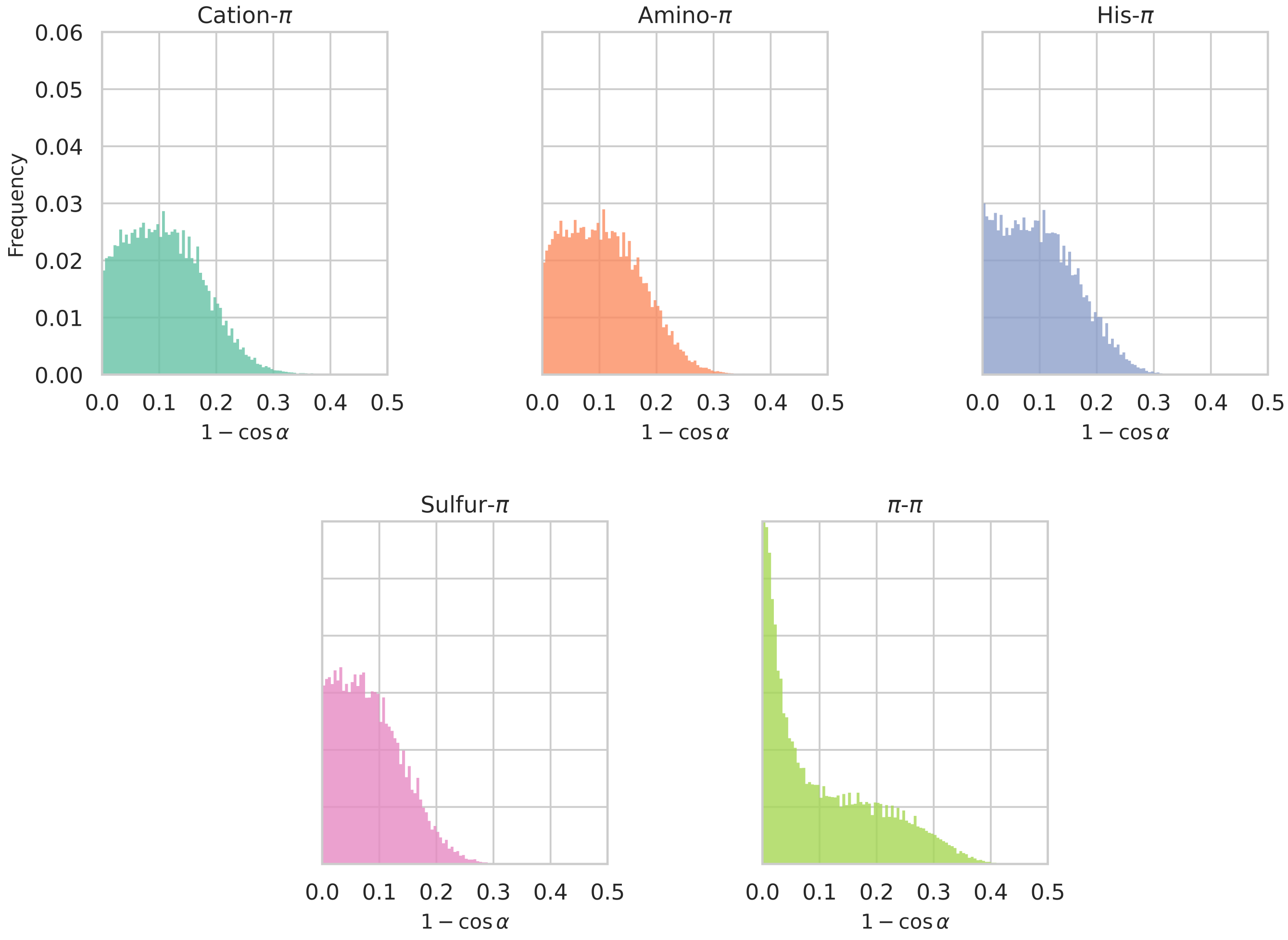

Figure S3: Distribution of the values of the lateral displacement with respect to the aromatic rings' center, $(1 - \cos\alpha)$, in which the α angle measures the location of the functional group of interacting partner 2 above (or below) the plane of the aromatic ring of partner 1, for different types of π interactions detected in the $\mathcal{D}_{\mathrm{AF}}$ dataset. The smaller the value of $(1 - \cos\alpha)$, the more directly the functional group of partner 2 is positioned above (or below) the center of the aromatic ring. This Figure must be compared with Figure 5 in the main manuscript that show the equivalent distributions in experimental structures.

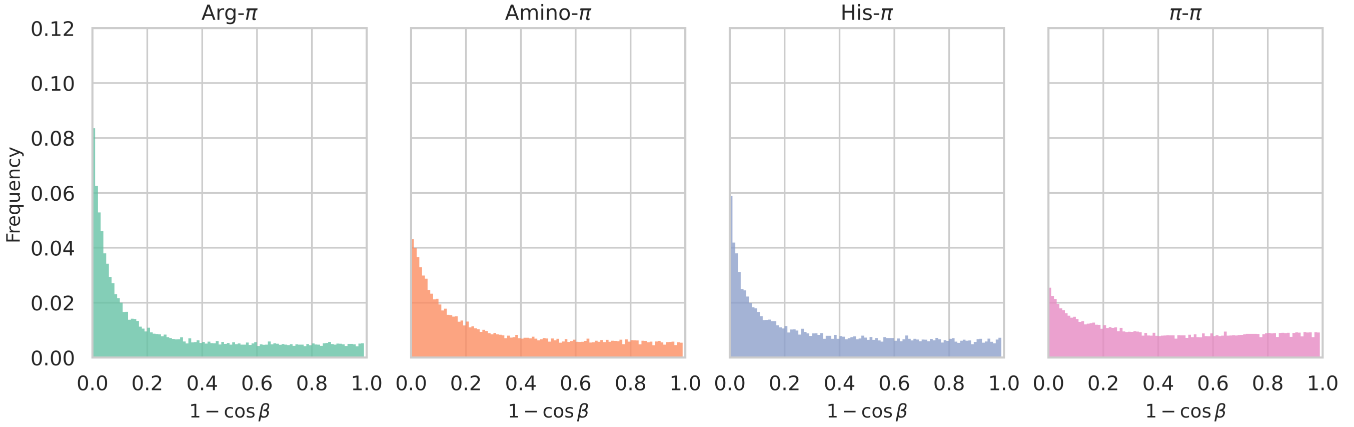

Figure S4: Distribution of $1 - \cos\beta$ values, in which the β angle measures the degree of parallelism between the interacting planar functional groups, for different types of π interactions detected in the $\mathcal{D}_{\mathrm{AF}}$ dataset. Parallel or stacked conformations correspond to $\beta = 0°$ and $1-\cos\beta = 0$; perpendicular or T-shaped conformations, to $\beta = 90°$ and $1-\cos\beta = 1$. This Figure must be compared with Figure 6 in the main manuscript that show the equivalent distributions in experimental structures.

Section S4. Example of π-π chains in amyloid fibrils

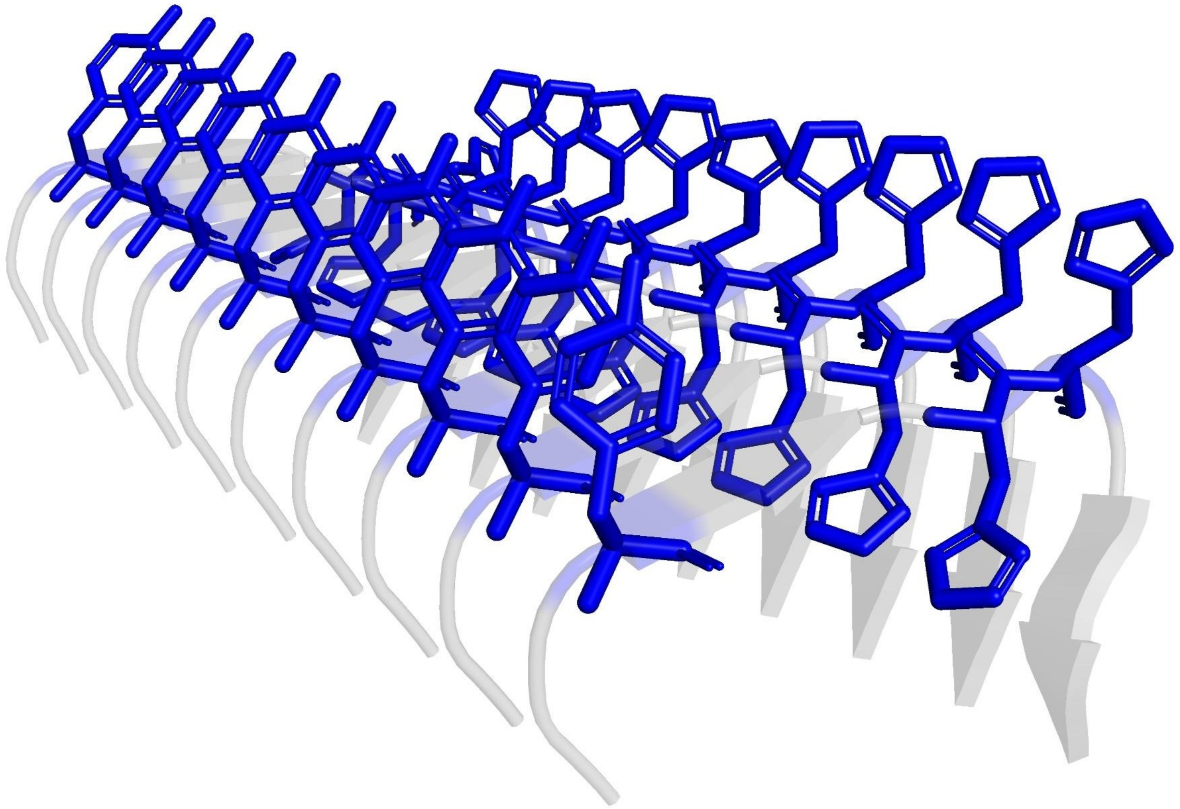

Figure S5: π-π Chain in an Aβ amyloid fibril purified from Alzheimer's brain tissue (PDB id: 6SHS) [58].

Section S5. Running efficiency of PInteract

Table S2: Running time of PInteract on datasets of different size.

Dataset	Number of samples	Time (seconds)
Arabidopsis thaliana	27,434	90.1
Escherichia coli	4,363	9.5
Homo sapiens	23,391	129.4
Saccharomyces cerevisiae	6,039	24.0
